# Mitochondria-related lncRNAs: predicting prognosis, tumor microenvironment and treatment response in lung adenocarcinoma

**DOI:** 10.1007/s10142-023-01245-3

**Published:** 2023-10-21

**Authors:** Qianhui Zhou, Jiali Xiong, Yan Gao, Rong Yi, Yuzhu Xu, Quefei Chen, Lin Wang, Ying Chen

**Affiliations:** Department of Respiratory and Critical Care Medicine, Zhuzhou Central Hospital, Zhuzhou, 412000 Hunan China

**Keywords:** Lung Adenocarcinoma, Mitochondrial, lncRNA, Prognosis model, Risk score

## Abstract

Lung cancer is the most common type of malignant tumor that affects people in China and even across the globe, as it exhibits the highest rates of morbidity and mortality. Lung adenocarcinoma (LUAD) is a type of lung cancer with a very high incidence. The purpose of this study was to identify potential biomarkers that could be used to forecast the prognosis and improve the existing therapy options for treating LUAD. Clinical and RNA sequencing data of LUAD patients were retrieved from the TCGA database, while the mitochondria-associated gene sets were acquired from the MITOMAP database. Thereafter, Pearson correlation analysis was carried out to screen mitochondria-associated lncRNAs. Furthermore, univariate Cox and Lasso regression analyses were used for the initial screening of the target lncRNAs for prognostic lncRNAs before they could be incorporated into a multivariate Cox Hazard ratio model. Then, the clinical data, concordance index, Kaplan–Meier (K-M) curves, and the clinically-relevant subjects that were approved by the Characteristic Curves (ROC) were employed for assessing the model's predictive value. Additionally, the differences in immune-related functions and biological pathway enrichment between high- and low-risk LUAD groups were examined. Nomograms were developed to anticipate the OS rates of the patients within 1-, 3-, and 5 years, and the differences in drug sensitivity and immunological checkpoints were compared. In this study, 2175 mitochondria-associated lncRNAs were screened. Univariate, multivariate, and Lasso Cox regression analyses were carried out to select 13 lncRNAs with an independent prognostic significance, and a prognostic model was developed. The OS analysis of the established prognostic prediction model revealed significant variations between the high- and low-risk patients. The AUC-ROC values after 1, 3, and 5 years were seen to be 0.746, 0.692, and 0.726, respectively. The results suggested that the prognostic model riskscore could be used as an independent prognostic factor that differed from the other clinical characteristics. After analyzing the findings of the study, it was noted that both the risk groups showed significant differences in their immune functioning, immunological checkpoint genes, and drug sensitivity. The prognosis of patients with LUAD could be accurately and independently predicted using a risk prediction model that included 13 mitochondria-associated lncRNAs.

## Introduction

Lung cancer is a malignant form of cancer that is associated with a shorter survival period and higher incidence rates. Lung cancer deteriorates quickly and is extremely dangerous, so if it is not treated in its early stages, it can threaten the patient's life (Imielinski et al. 2012). The data published by the World Health Organization (WHO) stated that 820,000 new lung cancer cases were reported in China in 2020, resulting in 710,000 deaths, which was equivalent to at least one death per minute due to lung cancer (Gao et al. 2020). LUAD is a type of non-small-cell lung carcinoma (NSCLC) that generally affects young women. The patients show no obvious clinical symptoms during the early stages of their disease, and tumor growth is generally slow. However, hematogenous metastasis can occur in the early stages, while lymphatic metastasis is more common during the later stages. Thus, LUAD is often diagnosed at advanced stages (Travis et al. 2011; Alexander et al. 2020).

Non-coding RNAs (NC-RNAs) are encoded by the genome but are not translated into proteins (Khalil et al. 2009). Though they are not translated, NC-RNAs are involved in many physiological and cellular functions (Guttman et al. 2009). It has been reported that lncRNAs (long NC-RNAs; length > GT; 200 NT) play a vital role in regulating cell growth, cell differentiation, gene expression, and cell development (Bhan and Mandal 2015). Studies have reported the presence of numerous abnormally-expressed or mutated lncRNAs in various cancers (Bhan and Mandal 2014). Furthermore, the onset and metastasis of the tumors can be attributed to aberrant expression, mutation, and single-nucleotide polymorphism of lncRNAs. It was noted that some of the lncRNAs behaved as oncogenes; whereas a few others played a tumor-suppressing role (Baldassarre and Masotti 2012). In the past, studies identified new abnormally expressed lncRNAs that could lead to the onset and progression of cancer. Furthermore, a lot of evidence has indicated that functional lncRNAs could be used as promising biomarkers and possible targets for cancer prognosis.

Mitochondria are involved in the cellular energy metabolism process, and abnormal mitochondrial function is an important cause of cell death or failure (Tait and Green 2012). It has been demonstrated that lncRNAs promote or inhibit the development of cancer by regulating mitochondrial function (Zhao et al. 2018a). In renal cell carcinoma, lncRNA MEG3 increased the cytochrome release by reducing BCL-2 expression and increasing CASPSAE-9 activity, leading to apoptosis (Wang, et al. 2015). In ovarian cancer, mitochondria-related lncRNA Gas5 was seen to promote apoptosis by reducing mitochondrial membrane potential after promoting the Bax and Bak expression and increasing the Caspase-3 and 9 activities (Kleih et al. 2019). In addition, mitochondria-related lncRNA also plays an important role in cellular senescence. For example, overexpression of lncRNA ASncmt RNA-2 induced the endothelium to exit the G1 phase and enter the G2M phase, thereby promoting cell proliferation, accelerating cellular proliferative senescence, and ultimately leading to vascular aging (Farfán et al. 2021). Thus, mitochondrial dysfunction was identified as the primary mechanism causing lung cancer, however, the changes occurring in the mitochondrial state in LUAD remain unknown.

Therefore, in this study, the data regarding the mitochondria-associated lncRNAs was acquired from the TCGA database and used to develop a LUAD prognosis model. The data were also employed to screen novel molecular biomarkers for LUAD prognosis, assess the patient prognosis, carry out functional enrichment analysis, and analyze their immune-related functions. Finally, the probable mechanism of LUAD was elucidated to offer novel insights and present a new direction for the targeted therapy.

## Materials and methods

### Acquiring the information related to LUAD patients

Herein, the RNA-seq transcriptome data of FPKM-normalized 501 LUAD tissues and 54 normal tissues were downloaded from the TCGA database. All the respective clinical data regarding the age, sex, survival period, survival status, histological grade, and TNM stage of the patients were also downloaded. The transcriptome data were sorted, ID transformed, and the lncRNAs were identified using the Perl software. To reduce statistical analysis bias, LUAD patients, who did not survive, were excluded from this study. All mitochondria-related genes were downloaded from MITOMAP.

### Selecting the mitochondria-related genes and LncRNAs

Mitochondrial-related genes and lncRNAs were identified for co-expression analysis using Pearson Correlation Analysis (|Pearson R|> 0.6 and *p* < 0.001). The differential expression of mitochondria-related lncRNAs was determined with the help of the combined DESeq2, EdgeR, and Limma software (Voom) [False Discovery Rate (FDR) of < 0.05 and Log2 Fold Change (FC) > 1].

### Developing and verifying the risk signature

After assessing the above data related to the LUAD patients, Univariate Cox regression analysis was conducted to determine the target lncRNAs linked to the prognosis of LUAD patients. The data were also screened using the Lasso regression analysis to reduce data overfitting, and the critical and mitochondria-related lncRNAs were identified. Lasso regression used cross-validation for parameter selection, and the spectrum of coefficients was plotted. Lastly, multivariate regression analysis was carried out to establish a mitochondria-related lncRNA model for LUAD prognosis and the data were presented using nomograms. The Risk score equation of lncRNAs based on multivariate regression analysis was as follows: Risk score = $\sum_{i=1}^{n}{cofficient}_{i}\times EXP{\left(lncRNA\right)}_{i}$. The novel prognostic model was used for estimating the risk score of every patient, and all LUAD patients were categorized into high-risk and low-risk groups based on their median risk value.

### Independence factors and ROC

The KM technique was employed to analyze the differences (variations) in the survival duration of the patients in the two risk groups and determine the prognostic role played by the risk score in LUAD patients. Univariate and multivariate Cox-independent prognostic analyses were conducted for determining the effect of sex, age, histological grade, risk scores, and clinical stages on the LUAD prognosis. This was conducted to assess if riskscore can be used as a novel and independent prognostic factor that was not based on other clinical features. Then, the predictive performance of the prognostic model was assessed using ROC.

### Nomogram and calibration

The RMS tool in R software was employed for data analyses. Here, all the data were packaged after combining the age, tumor stages, clinical data, T stage, and risk score data with the variable data of nomograms to develop a novel nomogram based on the nomogram function. Calibration diagrams were utilized for assessing the accuracy of these nomograms and validating their performance.

### Gene set enrichment analyses

RNA-seq expression profiles were utilized for gene set enrichment analysis (GSEA) for identifying the relevant signaling pathways associated with the differentially expressed genes (DEGs) in the two risk groups in the cohort. Statistical significance was established at FDR < 0.05 and *p* < 0.05.

### Evaluating the TME and immune checkpoint genes

The differences in the tumor immune microenvironment (TME) between the two risk groups were further examined using the "Maftools" R approach and GSEA results. The Wilcoxon signed-rank test was employed to compare the levels of immune cell infiltration, while the results were expressed using a bubble chart. Meanwhile, the immune, stromal, and estimate scores were calculated for describing the TME. Also, the variations in the expression levels of immune checkpoint genes in the two groups were analyzed.

### Exploring the model in drug sensitivity

The "Limma" and "GGPLOT2" tools of the R programming language were used to analyze the chemotherapeutic and targeted drug responses in LUAD patients. The half-maximal Inhibitory Concentration (IC50) can be used as an indicator of the anti-tumor activity of the drugs. This study analyzed the IC50 values of the targeted and chemotherapeutic drugs in both risk groups.

## Results

### Mitochondria-associated lncRNAs in LUAD patients

**Fig. 1:**
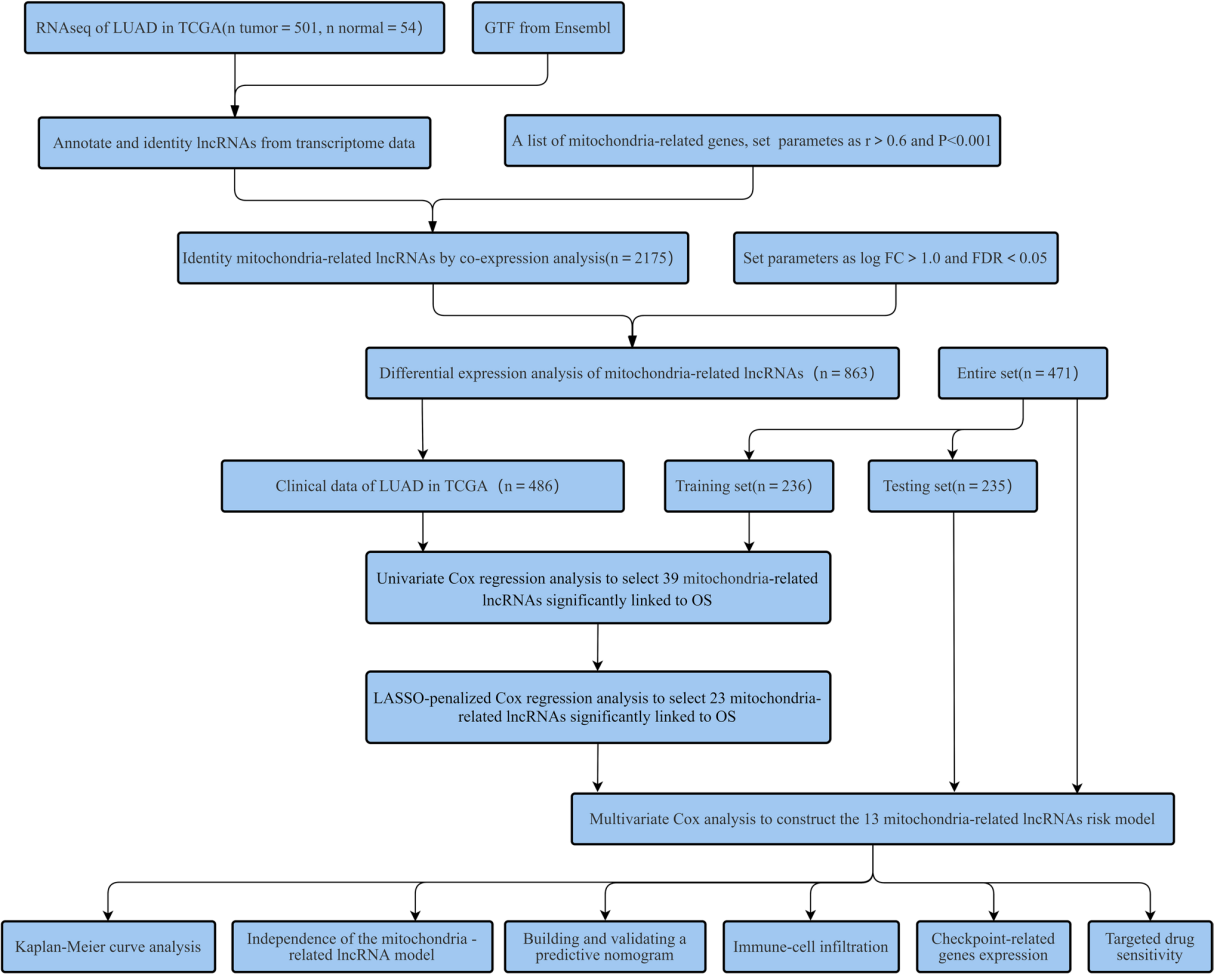
Flow chart depicting the experiments conducted in this study

**Fig. 2:**
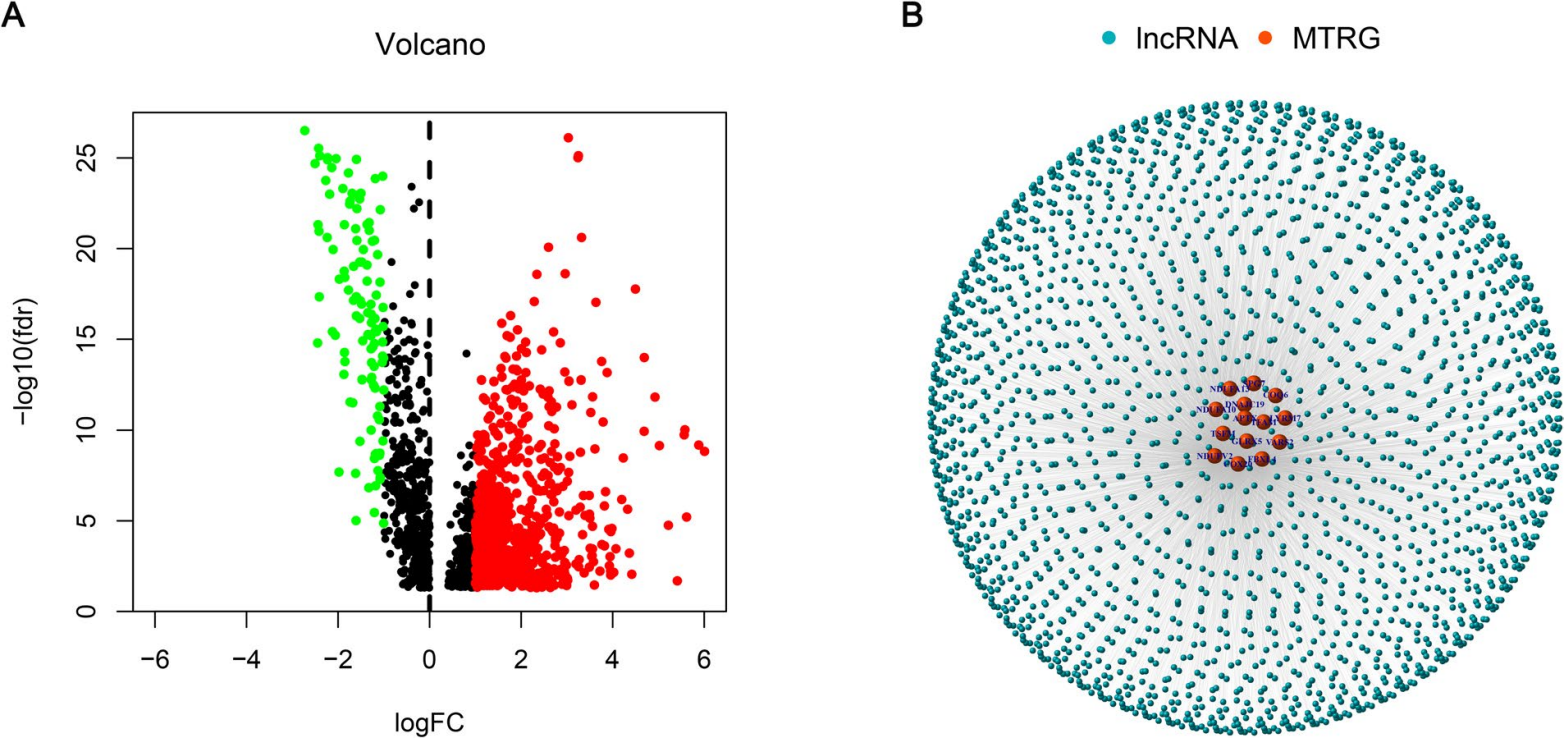
Mitochondria-related lncRNAs were extracted from LUAD patients. **A** Volcanic map of mitochondria-related lncRNAs showing a downregulated and upregulated differential expression. **B** The distribution network of mitochondrial genes and lncRNAs (correlation coefficient > 0.4 and *p* < 0.001)

Figure 1 presents the flow chart of all experiments and techniques used in the study. Initially, 501 malignant and 54 healthy samples were downloaded from the TCGA dataset. Then, Pearson correlation analysis was employed to detect 2,175 mitochondria-related lncRNAs (|Pearson R|> 0.6 and *p* < 0.001). Furthermore, Strawberry Perl and Limma R software were used to assess the DEGs between the malignant and healthy samples, and 863 mitochondria-related lncRNAs were screened. (Log2 FC > 1 and FDR < 0.05). Out of the 863 mitochondria-related lncRNAs, 751 were seen to be upregulated, whereas 112 were downregulated (Fig. 2A). Figure 2B presents the network and comparative data between the mitochondria-related genes (like COX20 and FBXL4), and lncRNAs.

### Constructing and validating the developed model

**Fig. 3:**
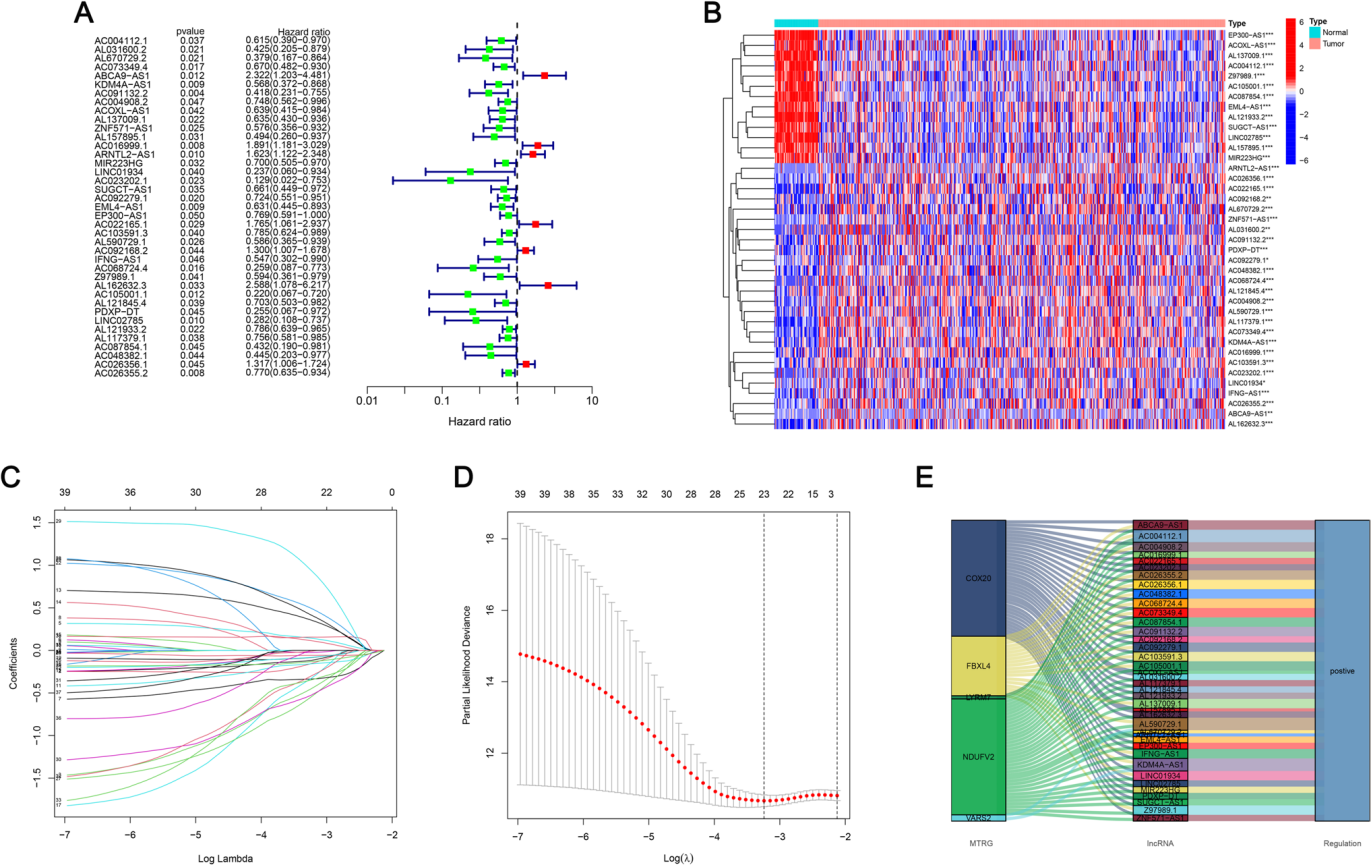
Construction of a novel prognostic model incorporating mitochondria-related lncRNAs in LUAD patients. **A** The forest plot shows the prognostic value of the lncRNAs using Univariate Cox proportional hazard analysis. **B** Gene heatmap of 39 mitochondria-related lncRNAs. **C** Lasso regression of the model was constructed based on the optimal parameters (Lambda). **D** Lasso regression coefficient curves of 39 mitochondria-related lncRNAs. **E** The Sankey diagram of lncRNAs associated with mitochondrial genes and prognosis

**Fig. 4:**
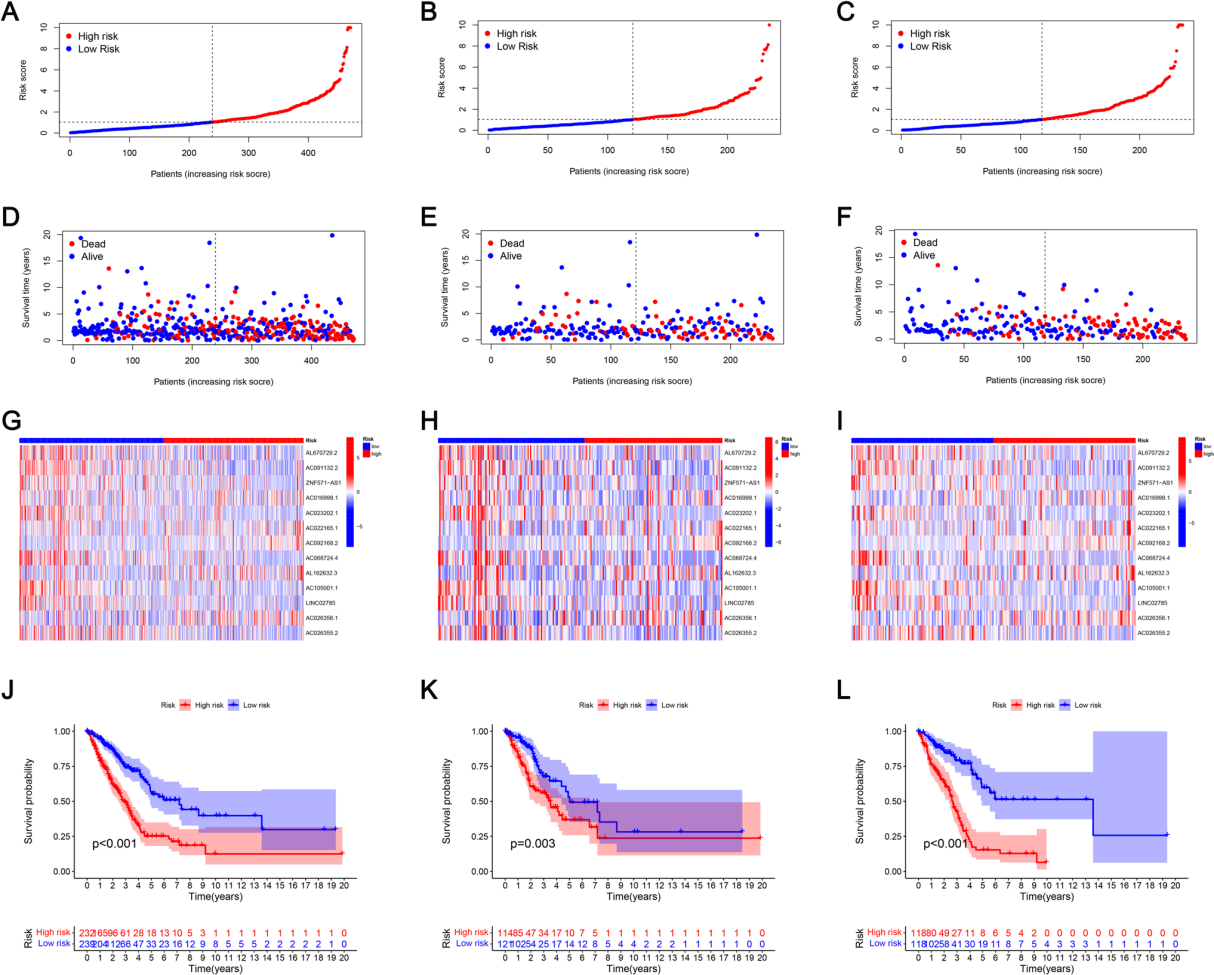
Prognostic value of 13 risk models associated with the mitochondria-linked lncRNA models in the training, testing, and entire sets. The risk score of the samples was distributed across the entire (**A**), training (**B**), and testing (**C**) sets. The red color in the diagram indicated the high-risk groups, while the blue color denoted the low-risk patients. A scatter plot of the survival time of each sample between both risk groups in the entire (**D**), training (**E**), and testing (**F**) sets. Heat map of the 13 lncRNA expression levels between both the groups in the training (**G**), testing (**H**), and entire (**I**) sets. KM survival analysis variations between both groups in the entire (**J**), training (**K**), and testing (**L**) sets

**Fig. 5:**
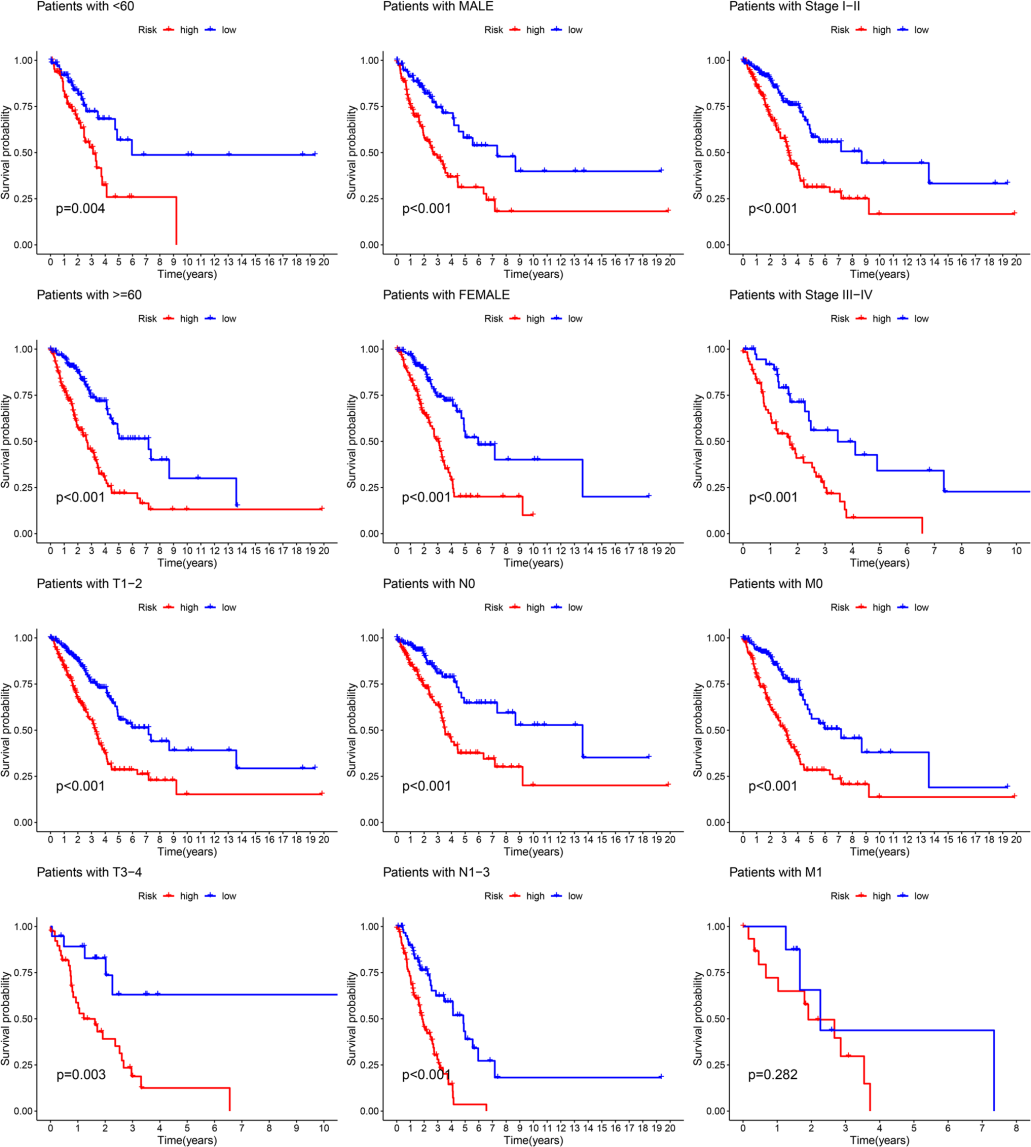
KM survival subgroup analysis was performed for the entire set, stratified by clinical features and mitochondria-related lncRNA features

Firstly, Univariate Cox regression analysis was conducted to detect 39 target lncRNAs for prognostic relevance in LUAD patients (Fig. 3A). Then, a heat map was generated (Fig. 3B) and presented as a Sankey plot (Fig. 3E). Thereafter, Lasso regression was conducted for screening the variables to minimize the data overfitting. The findings revealed 23 mitochondria-related lncRNAs that were quite effective for disease prognosis. Then, the Lasso regression coefficient spectrum (Fig. 3C) and cross-validation plots of parameters (Fig. 3D) were drawn. Subsequently, multivariate Cox regression analysis was conducted to screen 13 mitochondria-related lncRNAs that showed a significant prognostic value in LUAD patients. The riskscore of every LUAD patient was estimated using the riskscore formula. The survival variation between the two risk groups was analyzed based on the entire, training, and testing sets. After analyzing the results displayed by the risk score chart, OS status chart, and risk heat chart, it was concluded that an increase in risk score led to a gradual increase in the mortality rates of the LUAD patients. It was also noted that the OS rate in the low-risk LUAD group of patients was significantly better in comparison to the value in high-risk patients (Fig. 4A-L). In addition, the clinical characteristics of LUAD patients, such as sex, age, tumor stages, N stage, T stage, and M stage displayed similar results (Fig. 5).

### Construction of nomogram

**Fig. 6:**
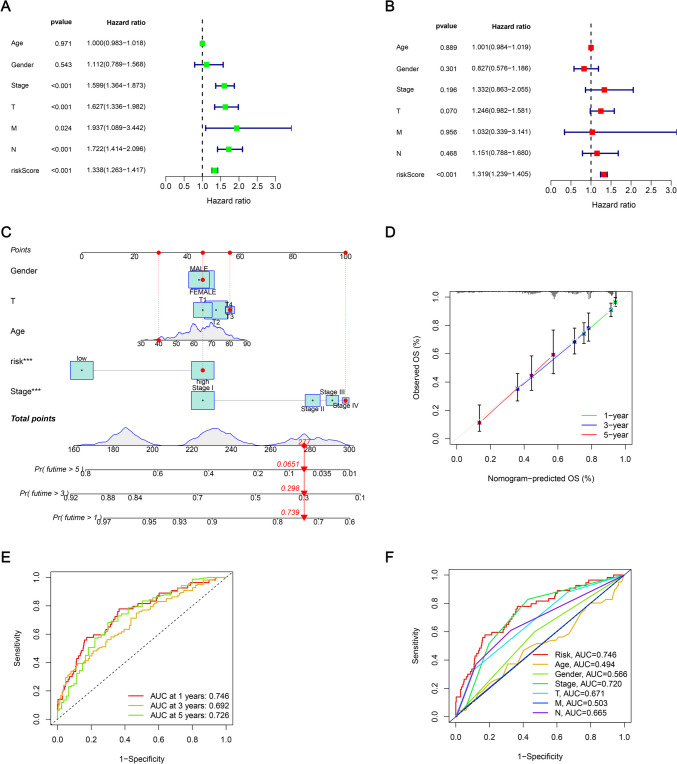
A nomogram integrating 13 mitochondria-related lncRNAs of risk model and clinical data was developed and verified. (**A**) Univariate and (**B**) Multivariate Cox analyses were carried out to validate the independence of the developed risk model in prognosis prediction. (**C**) A clinical prognostic nomogram was developed to predict 1-, 3-, and 5-year OS rates. (**D**) Calibration curves were plotted for predicting 1-, 3-, and 5-year OS. (**E**) 1-, 3-, and 5-year ROC curves for the entire set. (**F**) Time-dependent ROC curve analysis was used to predict 1-year OS using clinical factors like age, sex, risk score, stage, T stage, M stage, and N stage

To assess the clinical value of the developed prognostic model, univariate and independent multivariate prognostic analyses were carried out using the riskscore and clinical data (i.e., gender, tumor stages, age, T stage, N stage, and M stage). The Univariate Cox regression analysis revealed that the tumor stage, N stage, T stage, M stage, and total riskscore were all independent prognostic factors (Fig. 6A), while the Multivariate analysis implied that only riskscore was an independent prognostic factor (Fig. 6B). Risk scores were incorporated into clinical factors, the data was plotted, and survival nomograms were developed to predict the 1-, 3-, and 5-year OS values in LUAD patients (Fig. 6C). The results of the calibration chart indicated that the predicted probability was in agreement with the actual probability, which showed that the estimated and actual mortality rates were comparable (Fig. 6D).

### Assessment of the developed risk model

The ROC curve that was plotted for evaluating the 1-year survival prediction performance of the LncRNA prediction model showed an AUC = 0.746, while the ROC curves for the 3-year and 5-year survival prediction showed AUC values of 0.692 and 0.726, respectively (Fig. 6E). The AUC of the clinical prognostic nomogram was 0.746 for the 1-year ROC curve, which was seen to be significantly higher compared to clinical factors like age (AUC = 0.494), sex (AUC = 0.566), tumor Stage (AUC = 0.720), T Stage (AUC = 0.671), M Stage (AUC = 0.503), and N Stage (AUC = 0.665) (Fig. 6F). Then, the discriminant ability of the model to predict the survival rate of LUAD was further verified.

### Gene set enrichment analysis (GSEA)

**Fig. 7:**
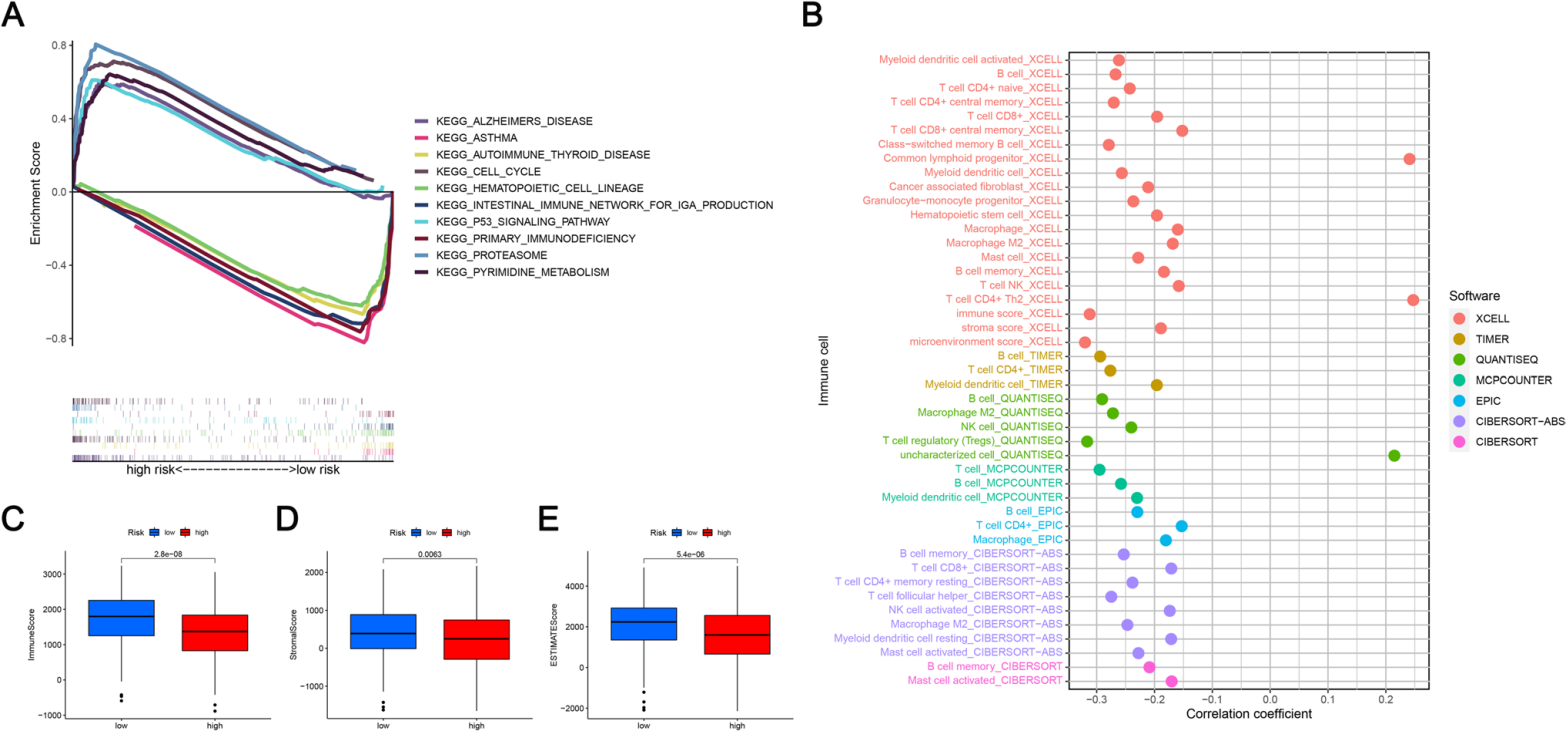
Investigation of TME and immunotherapy. **A** The top 5 KEGG signaling pathways in the low- and high-risk groups were assessed using GSEA. **B** The immune cell bubble for both risk groups. **C**-**E** Differences between immune stroma and immune cells in both risk groups. **C** Stromal score; **D** Immune score; **E** Estimate score

GSEA analysis was conducted in both risk groups to investigate the potential molecular mechanisms used by the 13 mitochondria-related lncRNA model in LUAD, and the KEGG pathway was identified. Figure 7A summarizes the top 5 KEGG pathways in the two cohorts. The results indicated that the top 5 KEGG pathways in the high-risk LUAD group were Alzheimer’s disease, p53 signaling pathway, Cell cycle, Proteasome, and pyrimidine metabolism, whereas the top 5 pathways in the Low-risk patients were Asthma, Autoimmune thyroid disease, Primary immunodeficiency, Hematopoietic cell lineage, and Intestinal immune network for IGA production.

### Analysis of differences in the immune microenvironment and clinical treatment in both the two groups

**Fig. 8:**
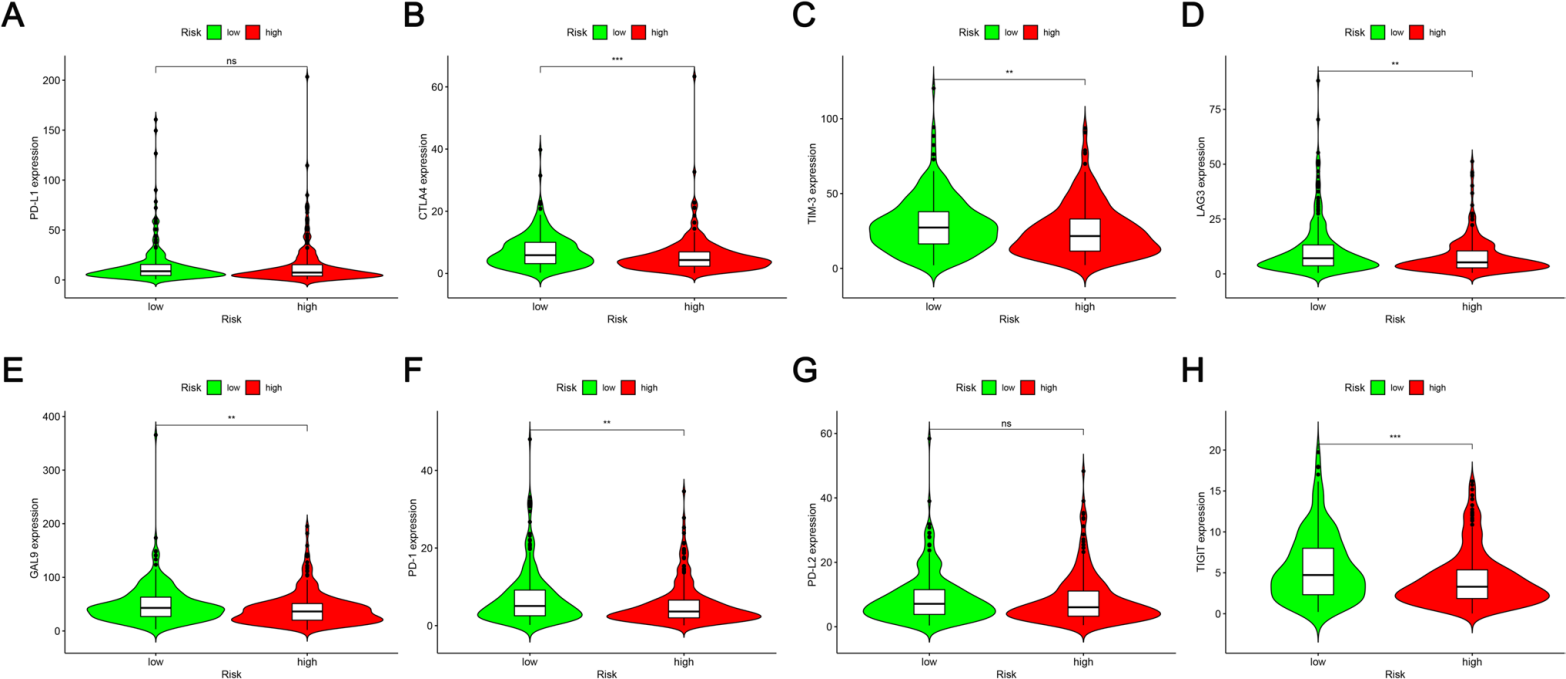
Differences noted in the expression levels of eight immune checkpoint genes across both risk groups

**Fig. 9:**
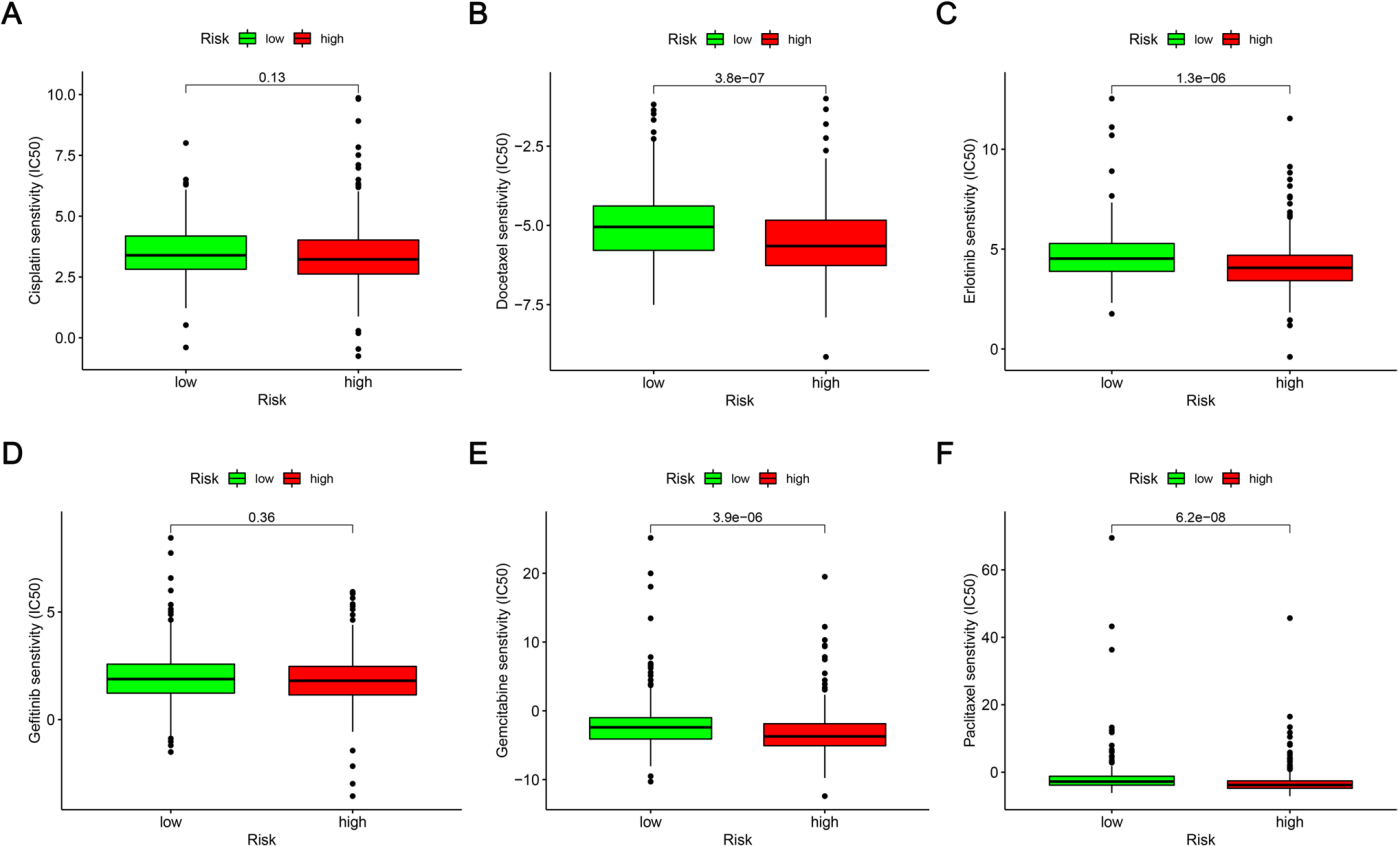
Six therapeutic drugs showing significant differences in their IC50 values

More immune cells were connected with the lower-risk category on several platforms, as shown in the immune cell bubble chart and document. These included the Common lymphoid progenitor and T cell CD4 + Th2 at XCELL, uncharacterized at QUANTISEQ (all *p* < 0.05) (Fig. 7B). Furthermore, the high-risk patients exhibited significantly low immune scores (Fig. 7C), stromal scores (Fig. 7D), and Estimate score values (Fig. 7E), which differed significantly from the TME displayed by the low-risk patients. The results indicated that the low-risk patients showed a low tumor purity in comparison to the high-risk LUAD patients. It was also noted that the eight immune checkpoint genes displayed a lower expression level in high-risk patients, out of which genes like CTLA4, TIM-3, LAG3, CAL-9, PD-1, and TIGIT showed a statistically significant difference between both risk groups (Fig. 8). These results implied that the low-risk LUAD patients had a very active immune system. It was also concluded that high-risk patients displayed a poor prognosis and a poor effect of immunotherapy. IC50 can be used as an indicator of the anti-tumor activity of drugs. This study analyzed the IC50 values of targeted drugs and chemotherapeutic drugs in the two groups. The findings of this analysis indicated that high-risk patients had a lower IC50 value of the six LUAD chemotherapeutic drugs and targeted agents. More importantly, patients with a low-risk score were seen to be very sensitive to antineoplastic drugs like erlotinib, paclitaxel, docetaxel, and Gemcitabine (Fig. 9). Thus, it could be concluded that mitochondria-related lncRNAs are potential predictors of drug sensitivity.

## Discussion

Lung cancer is regarded as a leading cause of cancer-related deaths across the globe (Malhotra et al. 2016). The pathogenesis of LUAD is not well understood and effective therapeutic approaches are lacking (Bade et al. 2020). In comparison to the single clinical biomarker, the integration of multiple biomarkers into one model improves predictive accuracy and facilitates the development of individualized treatment plans. Recently, second-generation sequencing technology has revolutionized the prognostic prediction of cancer (Sears and Mazzone 2020; Hirsch et al. 2017). In routine clinical practice, pathological staging is an important prognostic determinant of LUAD. However, many differences were observed in the clinical findings of same-stage patients, suggesting that traditional staging systems are unable to adequately predict patient outcomes. Hence, there is a considerable need to identify and develop biomarkers related to tumor diagnosis and prognosis.

Mitochondria are not only energy factories but are also involved in cell growth, differentiation, senescence, and different cell death processes (Bock and Tait 2020). In the past few years, many studies have highlighted the involvement of mitochondrial metabolism and mitochondrial dysfunction in the development of cancer (Srinivasan et al. 2017; Porporato et al. 2018). LncRNAs were recognized as effective biomarkers that were involved in the initiation and progression of different tumors like LUAD (Zhao et al. 2018b). Song et al. found that the mitochondria-related lncRNA, i.e., MDL1, controls nuclear gene expression by regulating the subcellular localization of the transcription factor (p53 protein), thus leading to a retrograde regulation of nuclear gene expression by the mitochondria (Li et al. 2022). LncRNAs are an emerging biomarker and a current research hotspot for non-coding RNAs, which are seen to be involved in cell life activities and play critical roles (Zhao et al. 2018b; Wei et al. 2020). LncRNAs can participate in cellular biological functions by exerting their endogenous “Mirna sponge” function and RNA target interaction (Batista and Chang 2013). Studies in the past have confirmed that the lncRNA expression was significantly up- or down-regulated and was involved in several malignant biological processes like invasion, proliferation, and apoptosis of tumor cells, and was also closely associated with drug sensitivity (Huang et al. 2017; Yang et al. 2022; Li et al. 2018). Some studies have identified a group of lncRNAs that could help in differentiating between the early LUAD tissue and normal lung tissue with high sensitivity and specificity. It is suggested that abnormally expressed lncRNAs may be used as a potential biomarker for diagnosing early-stage LUAD patients (Wang et al. 2015). Nevertheless, it is uncertain whether mitochondria-related lncRNA could be utilized to anticipate the prognosis of LUAD patients. In this study, a novel predictive model for LUAD was developed that displayed a better patient survival probability rate by screening for mitochondria-related lncRNAs.

In this study, 147 mitochondria-related genes and 2175 mitochondria-related lncRNAs were identified. Subsequently, 863 mitochondria-associated lncRNAs were identified by differential expression analysis. LUAD patients with complete clinical data were randomly classified into two different sets, i.e., training and testing sets. Then, univariate regression analysis was carried out in this study for identifying 39 mitochondria-related lncRNAs in the training set. Furthermore, LASSO regression was used for dimensionality reduction of the data to prevent overfitting. This yielded 23 mitochondria-related lncRNAs that were closely associated with OS in LUAD patients. Finally, 13 mitochondria-related lncRNAs were detected by performing multivariate Cox regression analysis, and a novel prognostic model was constructed. Some of these mitochondria-related lncRNAs have been reported in the past and were seen to be closely related to tumor initiation and progression. In their study, Wu et al. noted that AC092168.2 was a member of the immune-related-lncRNA prognostic signature of LUAD (Wu et al. 2021). Additionally, several studies revealed that AC026355.2 was engaged in a variety of processes, including immunomodulation, autophagy, pyroptosis, necroptosis, etc., which may have contributed to the onset and progression of LUAD (Lu et al. 2022; He et al. 2021; Liu et al. 2022; Gong et al. 2022). However, ZNF571-AS1 was first reported in solid tumors, and earlier studies have highlighted its role in different diseases such as dilated cardiomyopathy, acute myeloid leukemia, and Alzheimer's disease (Chen et al. 2021; Pan et al. 2017; Li et al. 2022). However, their specific mechanism of action in LUAD is not fully understood and needs further investigation. Very few studies have investigated the involvement of the remaining 10-mitochondria-related lncRNAs, and, although little is known about them, their importance should not be underestimated.

Furthermore, a prognostic model was developed to predict the survival of LUAD patients based on 13 mitochondria-related lncRNAs, which could be independently used as a prognostic marker for LUAD. The model accurately classifies the LUAD patients into two risk groups: low-risk and high-risk groups. The low-risk patients showed a better outcome in the training, testing, and entire sets. As shown in the results, both risk groups presented significant differences in their survival curves. The findings indicated that the high-risk score patients showed higher mortality rates in the training, testing, and entire sets. Furthermore, it was noted that an increase in the risk score led to a subsequent increase in the probability of patient death and a decrease in their survival time. On the other hand, low-risk patients showed a longer survival time. The training set data could therefore be used to construct a model based on 13 mitochondria-related lncRNAs, and it could accurately identify the prognosis of the patients and display good predictive power for the prognosis of LUAD patients.

In clinical practice, the tumor stage is determined by the tumor size, node, and metastasis (TNM) method, which is generally used for evaluating the prognosis of tumor patients. With the advent of precision medicine, an increasing number of studies have suggested that lncRNAs could have some predictive significance for tumor prognosis (Bhan et al. 2017; Lv et al. 2022; Yan et al. 2021). Therefore, the findings revealed that the prognostic model constructed by combining lncRNAs with tumor stage showed a higher predictive accuracy compared to the currently available methods for predicting prognosis. Nomograms are an easy-to-understand prognostic prediction model that is easy to operate, presents a high prediction accuracy, and is increasingly being used in medical research and clinical practice (Balachandran et al. 2015). In this study, a nomogram was constructed by integrating several clinical factors like age, TNM stage, and 13-mitochondria-associated lncRNA models based on the independent predictors generated by multivariate regression analysis. The ROC curves showed that nomograms were better than the individual prognostic factors (such as tumor stage) in predicting the disease prognosis. These results suggest that nomograms constructed based on the 13-mitochondria-associated lncRNA model may have more reliable clinical applicability and improve the accuracy of predicting prognosis.

Owing to differences in the genetic composition of the patients, targeted therapy can become an accurate and personalized treatment strategy. Identification of the molecular pathways related to LUAD could help in discovering new therapeutic targets. Therefore, GSEA was employed to screen for signaling pathways associated with 13 mitochondria-related lncRNA models, and the results indicated that these signaling pathways were associated with several cellular life activities. The P53 signaling pathway is closely associated with apoptosis and progression of the lung cancer cells (Wang, et al. 2019) and it is seen to regulate the immune responses (Muñoz-Fontela et al. 2016). Hence, the 13-mitochondria-associated lncRNA model that was constructed in this study was seen to be involved in a few cancer-related signaling pathways.

The lack of knowledge regarding the complexity, heterogeneity, and immune evasion mechanisms of tumors is one of the main challenges that affect the development of immunotherapy strategies for LUAD patients. In addition, there is a lack of specific biomarkers used to assess the benefit of tumor immunotherapy. Consequently, it is crucial to identify new immunotherapy targets and prognostic markers (Galon et al. 2014). The combination of immunosuppressive drugs can be regarded as an effective approach for treating several malignancies, and the activated TME is associated with a good response to immune checkpoint inhibitors (Yang 2015; Bersuker et al. 2019). The low-risk patients expressed high levels of the eight immunological checkpoints determined in this study, suggesting that these patients could be more benefitted by the use of immunosuppressive drugs. It was further indicated that the prognostic model developed in this study could help in predicting the effectiveness of immunosuppressive therapy.

Molecular targeted therapy has improved the treatment of lung cancer. The first molecularly-targeted therapeutic drug, i.e., gefitinib, has increased the survival duration of NSCLC patients by two times (Sun et al. 2020). Gefitinib and erlotinib, which are seen to be two small-molecule first-generation EGFR tyrosine kinase inhibitors (EGFR-TKI), were approved more than a decade ago and have been popularly used as a first-line treatment option for advanced NSCLC (Gelatti et al. 2019). The IC50 values of targeted drugs and chemotherapeutic drugs such as gefitinib, erlotinib, cisplatin, etoposide, paclitaxel, docetaxel, and Gemcitabine were analyzed using the pRRophetic tool. The findings suggest that high-risk patients may be more sensitive to chemotherapy and targeted therapy and could be used to determine the efficacy of targeted therapy in LUAD patients. These findings have potential implications for guiding the treatment and prognostic assessment of LUAD patients and may help in describing the relationship between the model and the drug to more accurately guide subsequent targeted drug therapy.

In conclusion, our study was a retrospective analysis based on the TCGA data set. It used a single sample source and could display a probable bias in the analysis results. This study on mitochondria-related lncRNAs lacks clinical and experimental validation, but future experiments would be conducted on gene expression and lncRNA function. Despite the aforementioned drawbacks, developing LUAD prediction models based on mitochondria-related lncRNAs may help in predicting the OS of LUAD more accurately than conventional pathological staging. This model could help in screening the high-risk LUAD population, and provide important references for the individualized treatment of all identified people. This would improve the research direction and offer a theoretical basis for follow-up clinical work and experimental development.

## Data Availability

The datasets generated and/or analyzed during the current study are available in the [https://portal.gdc.cancer.gov/].
